# Machine learning-driven microwave system for noninvasive monitoring of intracranial pressure

**DOI:** 10.1186/s12938-025-01453-x

**Published:** 2025-10-09

**Authors:** Daljeet Singh, Mariella Särestöniemi, Teemu Myllylä

**Affiliations:** 1 Research Unit of Health Sciences and Technology, Faculty of Medicine, University of Oulu, Oulu, 90570 Finland; 2 Infotech Oulu, Oulu, 90570 Finland; 3 Centre for Wireless Communications, Faculty of ITEE, University of Oulu, Oulu, 90570 Finland; 4 Opto-electronics and Measurement Technique Research Unit, Faculty of ITEE, University of Oulu, Oulu, 90570 Finland; 5 Medical Research Center (MRC), Oulu, 90570 Finland

**Keywords:** Brain monitoring, Flexible antenna, Intracranial pressure, Non-invasive sensing, Microwave imaging, Microwave medical sensing (MMS), Machine learning

## Abstract

Intracranial pressure (ICP) refers to the pressure inside the skull. It is influenced by the complex interactions between the volume of brain tissue, cerebrospinal fluid (CSF), and blood. Maintaining a normal ICP is crucial for normal brain function, as elevated ICP can restrict blood flow to the brain, potentially resulting in severe health issues. Because of this, there is significant interest in non-invasive methods for monitoring ICP. In this paper, a Machine Learning (ML) driven, non-invasive, and quantitative microwave method and setup are proposed for ICP monitoring in human subjects. The proposed method is independent of the type of microwave sensors and is carefully devised for accurate measurements based on two-level feature extraction, including advanced signal attributes. Six thin, small, lightweight microwave sensors are evaluated with different placement strategies for accurate ICP monitoring. The proposed method was tested on a realistic human phantom model developed exclusively for this study. The phantom model corresponds to the dielectric properties and hydrodynamics of a human head. A unique data set creation module and Ordered Selection Scheme (OSS) are also proposed to ensure real-time operation with a lightweight ML algorithm. In addition, the quantitative method is devised using weighted regression on signal attributes selected from OSS. It is deduced from numerous trials that the proposed microwave system can even detect minute changes in ICP, and its response is analogous to pressure values measured by invasive sensors used as a ground-truth device. The proposed microwave-based setup is potentially suitable for wearable applications, enabling safe and prolonged usage.

## Introduction

The temporal and spatial dynamics of intracranial pressure (ICP) provide critical diagnostic insights into overall brain health and function. The human brain is enclosed within the skull, which regulates the ICP by maintaining an optimal balance of its contents and is commonly defined by the Monro–Kellie doctrine. ICP is primarily controlled by the cerebral autoregulation system. Any abnormal change in the volume of brain tissue, cerebrospinal fluid (CSF), and blood can lead to dysregulated ICP, which is a key indicator of various neurological conditions [1, 2].

The invasive methods of ICP monitoring (catheter, external ventricular drain, or lumbar puncture) are susceptible to complications, such as infections, leakage, blockage, and misplacement errors. In addition, they carry risks of hematoma and ventriculitis, limiting their use for sensitive clinical cases and making them unsuitable for long-term use outside the hospital. Moreover, the use of invasive methods may be frightening for the patient and requires high expertise by the clinical personnel. To address the challenges associated with tethered devices, various microtransducer-based sensors have been developed [3]. Compared to fully invasive techniques, the subdural placement of these sensors minimizes infection risk and simplifies procedural complexity. However, many of these devices exhibit minor baseline drift and lack of in vivo calibrations. Moreover, these sensors induce pressure on the fontanelle, thereby altering the actual ICP. The procedural complexity of these techniques diminishes the clinical utility of ICP data in assessing brain health and function, lowering their use outside of neurocritical care [4–6].

Recent studies have demonstrated that ICP measurements not only reflect overall brain health but also provide insights into its functional efficiency, thereby necessitating frequent and prolonged monitoring, which is not possible by invasive techniques [7]. To overcome the limitations of invasive methods, several non-invasive techniques have been explored, including Optic Nerve Sheath Diameter ultrasound (ONSD) [8], Magnetic Resonance Imaging (MRI) [9], time-of-flight (ToF) ultrasound [10], transcranial Doppler (TCD) [11], near-infrared spectroscopy (NIRS) [12], and tympanic membrane displacement [13]. ONSD is portable and cost-effective but provides only indirect estimates and is operator-dependent. MRI offers high-resolution structural information but is bulky, costly, and unsuitable for continuous monitoring. ToF ultrasound, TCD, NIRS, and tympanic membrane displacement rely on surrogate markers and have limited temporal resolution [14]. In comparison, microwave-based systems offer the potential for compact, cost-effective, and real-time ICP monitoring. These systems explore various antenna designs, signal acquisition strategies, and feature extraction methods to capture ICP-related changes.

The propagation of microwave signals through biological tissue is governed by the dielectric properties of the tissue, specifically its permittivity and conductivity. These properties vary significantly across different tissues in the human head, including skin, skull, CSF, and brain tissue, resulting in distinct dielectric profiles. This dielectric contrast influences microwave propagation characteristics, such as the frequency-dependent spectral content and penetration depth of the signal. Changes in ICP are induced by volumetric changes in the head, which modify the effective dielectric profile, thereby altering microwave signal propagation. The proposed microwave system is designed and optimized to detect changes in signal propagation that reflect variations in ICP.

Chen et al. [15] developed a 1 cm$^3$ implantable passive sensor with wireless data transfer capability for ICP monitoring. The system was tested on mice for continuous pressure mapping using wireless frequency shift detection via inductive coupling. Redzwan et al. [3] presented a split-ring resonator as an implantable sensor and a microwave readout sensor for ICP monitoring, working on the same principle as [15]. An electromagnetic skin patch sensor has been developed in [16] for non-invasive ICP monitoring. The sensing mechanism in this study involves a shift in the resonant frequency of the sensor with volume change. A shift of 4.97 MHz was observed for a 200 ml increase. The sensor has large dimensions of 11.83 cm $\times$ 5.92 cm, which makes it unsuitable for practical use. Another skin patch sensor design was proposed by Griffith et al. with dimensions 51 mm $\times$ 56 mm [17]. The sensing mechanism of [16] was utilized in this study.

Perez et al. proposed a square spiral resonator coupled loop antenna for non-invasive ICP monitoring [18]. The study is based on phantom-based antenna reflection measurements. An inductive coil sensor based on simulations is proposed in [19], wherein the impedance of the sensor changes with blood volume. In this study, numerical simulations were performed for 100 MHz, 500 MHz, and 1 GHz, and the sensing mechanism involves tracking variations in amplitude and phase of the scattering parameters of the sensor. A coil-based sensor for cerebral blood flow monitoring is presented by Zhang et al. in [20]. The radius of the coil is 6 cm and has a resonant frequency of 8 MHz. An inductive sensing mechanism based on tracking changes in impedance and phase shifts of the sensor response is utilized. Recently, Singh et al. developed a microwave setup using flexible microstrip patch antennas designed on flexible Rogers5880 substrate for ICP monitoring [21]. The setup was tested on a realistic phantom model, and ICP was characterized using a shift in the magnitude of microwave signal reflection and transmission coefficients.

While promising results have been reported in phantom and preclinical experiments, most of the existing literature is based on an inductive sensing mechanism requiring a passive sensor to be implanted. In addition, the decision-making is based on manual tracking of sensor response corresponding to ICP changes, which is inefficient and cumbersome. This brute force approach corresponds to analyzing all antenna transmission ($S_{XX}$) and reflection ($S_{XY}$) coefficients for each time and frequency instance. Contrary to this, a Machine Learning (ML) driven microwave system is proposed in this study for noninvasive monitoring of ICP. The proposed system utilizes a two-level feature-based approach, wherein the decision-making is based on key features extracted from antenna response. The devised method is evaluated on a realistic head phantom using six microwave sensors. The proposed system is optimized in terms of required bandwidth, inter-antenna distance, and suitable ML-based regression models for a particular microwave antenna. For enhanced robustness, a unique Ordered Selection Scheme (OSS) and a data set creation module are proposed. The key contributions of this work are as follows:

- An ML-powered quantitative method and microwave system are proposed for the accurate monitoring of ICP based on two-level feature extraction, including advanced signal attributes. The devised method is independent of the microwave sensor and can be used for any microwave antenna.
- A data set creation module is devised to improve the robustness of the proposed quantitative method.
- A unique Ordered Selection Scheme (OSS) is proposed for feature selection, thus creating a lightweight algorithm ensuring real-time operation.
- Six microwave sensors are evaluated with different placement strategies for accurate ICP monitoring. These sensors are thin, small, planar, lightweight, and suitable for use in wearable devices and for prolonged usage.
- An exclusive phantom model precisely mimicking the biological properties of the human head is developed for testing the proposed system.

A distinctive feature of this ICP monitoring method is that the features are extracted from three parameters: change in amplitude, resonant frequency shift, and phase variations of sensor response for estimating the pressure. The measurement results obtained from extensive trials on realistic experimental setups show that amplitude change is dominant in estimating the pressure changes inside the brain, followed by phase variations. In contrast, the resonant frequency shift is only suitable for measuring large ICP changes. Overall, a strong analogy is observed between the proposed sensor response and the actual ICP measured by invasive sensors. The correlation analysis applied to measured signals after pre-processing showcases a very high correlation between true pressure values ($p_T$) from invasive sensors and statistical parameters from microwave sensors. The ML-powered regression analysis further validates this observation. These regression models are trained in a supervised manner using invasive ICP measurements as labels. The trained models are further tested on test data sets and evaluated using standard performance matrices to select an optimized regression model for a particular microwave sensor.

## Results and discussion

**Fig. 1:**
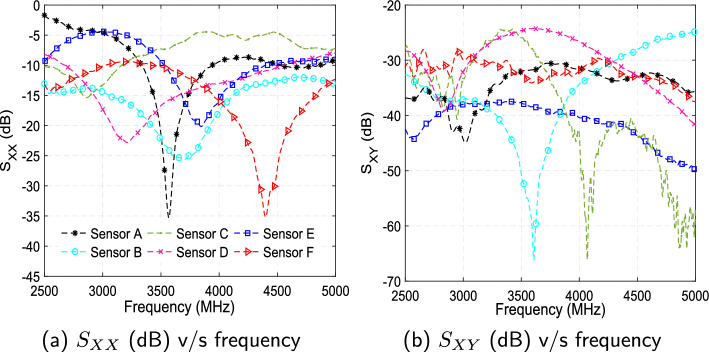
Input reflection coefficients ($S_{XX}$) and reverse transmission coefficients ($S_{XY}$) of the six microwave sensors v/s frequency

To enforce repeatability in measurements and reduce systematic errors, five independent trials are conducted for each measurement case presented in this study. The values from these five trials are averaged to produce the final results. The input reflection coefficients ($S_{XX}$) and reverse transmission coefficients ($S_{XY}$) of six microwave sensors when placed on the head phantom model are shown in Fig. 1a, b, respectively. It can be visualized from Fig. 1a that the frequency response of Sensor A has a sharp dip of $-$35.3 dB at 3.565 GHz ($S_{XX}$), while the curve for reverse transmission coefficients has a value $-$31.84 dB at this frequency. Sensor B has a comparatively wide frequency response encompassing the complete bandwidth of 2.5–5 GHz for $S_{XX}$ with $-$25.31 dB at 3.658 GHz, which is represented by the cyan line with a circle as a marker. The $S_{XY}$ plot for Sensor B in Fig. 1b shows a sharp dip of $-$65.87 dB at 3.604 GHz. The $S_{XX}$ response of Sensor C is relatively flat between -5 and -15 dB with a shallow dip at 2.886 GHz, suggesting moderate matching. Its $S_{XY}$ response is around -35 dB between 2.5 and 4 GHz, which drops to -50 dB from 4 to 5 GHz. Sensor D features resonance in $S_{XX}$ at 3.21 GHz ($-$22.83 dB) with operational BW of 2.6$-$4.6 GHz. The $S_{XY}$ response is stable around -35 dB. Sensor E shows resonance in $S_{XX}$ around 3.8 GHz (-20 dB), with $S_{XY}$ exhibiting attenuations around -40 dB throughout the entire operational BW. The $S_{XX}$ response of Sensor F is shown with a red line (triangle marker) in Fig. 1a, which exhibits a wide bandwidth with $S_{XX}$=-35 dB at 4.4 GHz. The $S_{XY}$ plot shows a stable response around -30 dB across the whole BW. The comparative analysis indicates that while all sensors operate within the designed frequency range, Sensor A and F achieve the deepest resonance dip in $S_{XX}$, indicating improved impedance matching. Sensors A, D, and F have wide operation bandwidths ($S_{XX}$ below -10 dB). Sensor B and C showcase strong $S_{XY}$ suppression ($\approx$ –65 dB), highlighting superior rejection capabilities.

### Correlation analysis

**Fig. 2:**
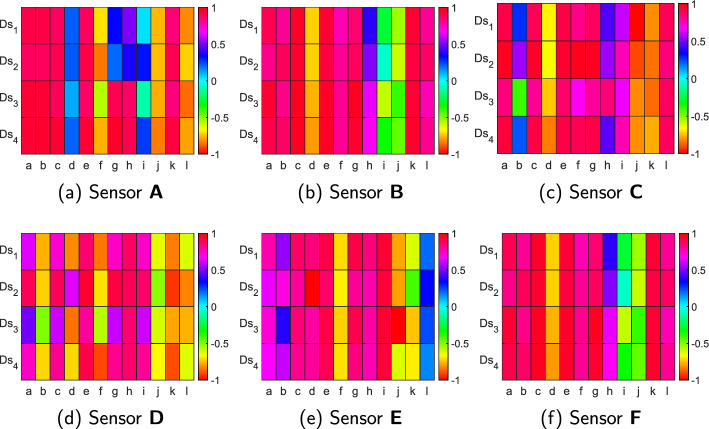
Heat maps of correlation between measured ICP (${{{\varvec{p}}}}_T$) from invasive sensors and features extracted from proposed system for Sensor A-F with $d_A$ = 2 cm. *a*:$min[|{{{\varvec{S}}}}^{i,j,f}_{XX}|]$, *b*:$min[|{{{\varvec{S}}}}^{i,j,f}_{XY}|]$, *c*:$AUC[|{{{\varvec{S}}}}^{i,j,f}_{XX}|]$, *d*:$AUC[|{{{\varvec{S}}}}^{i,j,f}_{XY}|]$, *e*:$AUC[GDD_{\angle {{{\varvec{S}}}}^{i,j,f}_{XX}}]$, *f*:$AUC[GDD_{\angle {{{\varvec{S}}}}^{i,j,f}_{XY}}]$, *g*:$AUC[MDM_D]$, *h*:$AUC[MDM_{RMS}]$, *i*:$PerAF_{|S_{XX}|}$, *j*:$PerAF_{|S_{XY}|}$; *k*:$PerAF_{\angle {{{\varvec{S}}}}^{i,j,f}_{XX}}$, *l*:$PerAF_{\angle {{{\varvec{S}}}}^{i,j,f}_{XY}}$

The correlation results of trials performed on realistic phantom models described in for different microwave sensors with $d_A$=2 cm are shown using heat maps in Fig. 2. All correlation values reported in this study are found statistically significant with $p < 0.001$, confirming the reliability of the observed relationships between the extracted microwave features and true ICP values. The rows of heatmaps in Fig. 2 showcase the four data sets, and the features are shown using columns. The correlation between true ICP values (${\varvec{{{p}}}}_T$) calculated by invasive sensors and features extracted from the proposed system showcases exciting trends. The heat maps of redundant and insignificant correlation values are removed from Fig. 2 due to size restrictions and to improve readability. Thus, the heatmaps showcase only the features selected through the proposed OSS scheme. It can be visualized from Fig. 2a that $min[|{\varvec{{{S}}}}^{i,j,f}_{XX}|]$ showcases high correlation values of 0.9095, 0.8779, 0.9354, and 0.9414 for ${\textbf {Ds}}_1$, ${\textbf {Ds}}_2$, ${\textbf {Ds}}_3$, and ${\textbf {Ds}}_4$, respectively. Similarly, $min[|{\varvec{{{S}}}}^{i,j,f}_{XY}|]$, denoted by column *b* and other features also have high correlation with ${\varvec{{{p}}}}_T$. Another interesting observation from columns *g* and *h* of Fig. 2a representing correlation of $AUC[MDM_D]$, $AUC[MDM_{RMS}]$, respectively, with ${\varvec{{{p}}}}_T$ is that ${\textbf {Ds}}_1$, ${\textbf {Ds}}_2$ bears relatively less correlation values when compared to ${\textbf {Ds}}_3$ and ${\textbf {Ds}}_4$. In some trials, the initial few samples may not be captured properly, producing such ambiguous behavior. The proposed data set creation module tackles this ambiguous behavior.

**Fig. 3:**
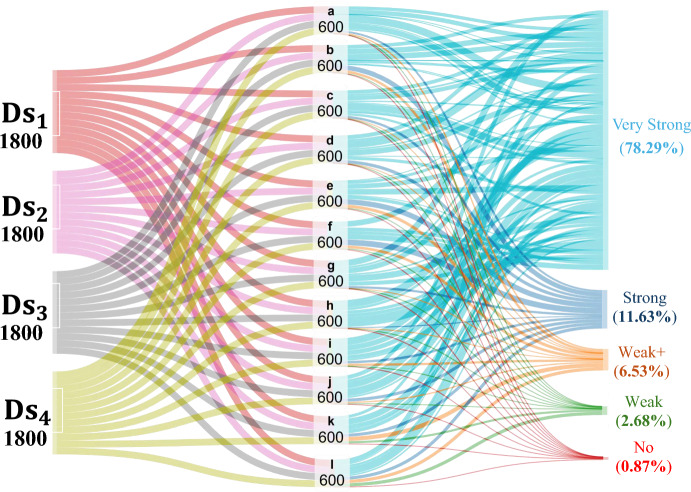
Sankey diagram of transition between different stages of quantitative ICP monitoring process, including data set creation, feature extraction, and correlation analysis

**Fig. 4:**
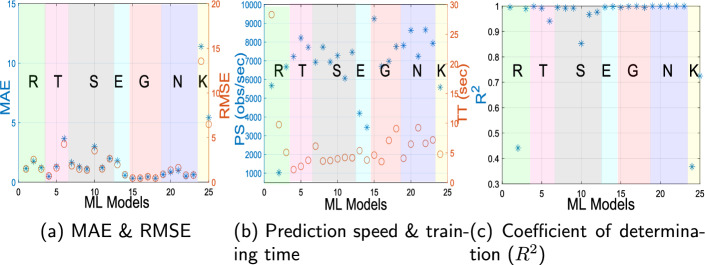
Performance comparison of different ML algorithms in ICP monitoring using Sensor A: (**a**) MAE v/s RMSE, (**b**) Prediction Speed (obs/sec) v/s Training Time (sec), (**c**) coefficient of determination ($R^2$); regression models: R: linear regression, T: tree, S: SVM, E: ensemble, G: Gaussian process regression, N: neural network, K: kernel-based regression model

**Fig. 5:**
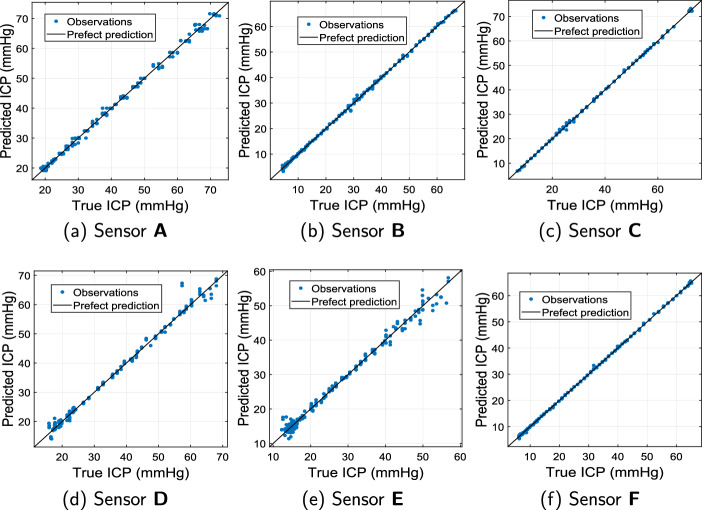
Plot of predicted ($\hat{p}_T$) v/s true ICP (${\varvec{{{p}}}}_T$) from ML models showcasing observations compared to perfect prediction for proposed microwave sensors **A**-**F** with $d_A$= 2 cm

**Fig. 6:**
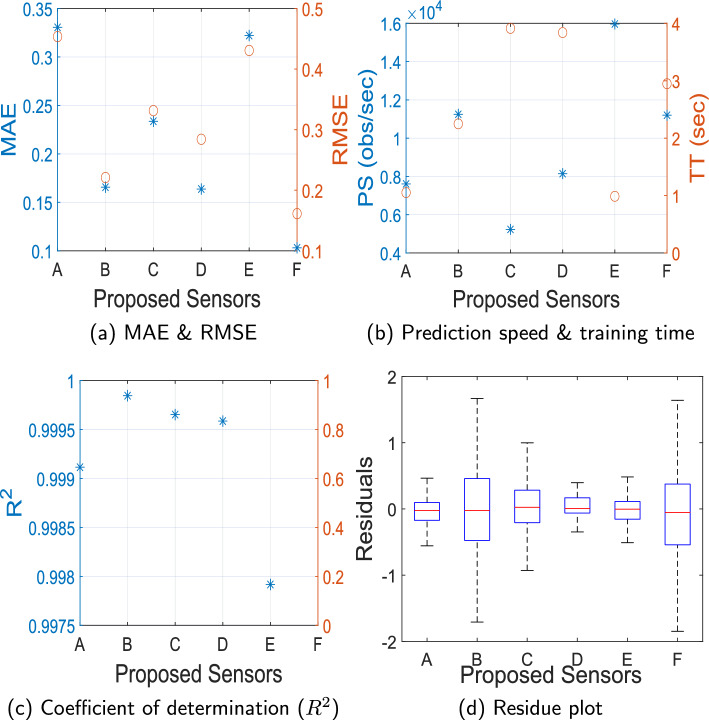
Performance comparison of different microwave sensors for ICP monitoring using optimized ML algorithm: (**a**) MAE v/s RMSE, (**b**) Prediction Speed (obs/sec) v/s Training Time (sec), (**c**) Coefficient of determination ($R^2$), (**d**) Residue plot of different ML models with proposed microwave sensors

To further study the behavior of the proposed system, the results of different sensors with all antenna placement strategies are plotted using a Sankey diagram in Fig. 3. A Sankey diagram shows the flow of a property or quantity between different states [22]. For each sensor, 5 trials are undertaken in each antenna configuration: $d_A$ = 0.5, 1, 2, 3 cm, and antenna on opposite sides, thus generating 25 trials. Apart from that, trials for testing and validation data sets are also conducted. Next, 100 data sets for one sensor are generated from these trials, and 1200 features are extracted. Therefore, for the six proposed sensors, a total of 7200 features representing 12 feature classes are extracted. It can be visualized from the correlation results in Fig. 3 that the proposed system showcases promising results for ICP monitoring. Out of the total 7200 test cases, 78.29% gives a very strong correlation (>0.8) with actual pressure (${\varvec{{{p}}}}_T$), and 11.63% gives a strong correlation ($\le$ 0.8 & > 0.6). Only 6.53% of cases showcases moderate correlation represented by weak+ ($\le$ 0.6 & > 0.4), and 2.68% of cases show weak correlation ($\le$ 0.4 & > 0.2). Furthermore, in only 0.87% of the cases, there is no correlation (< 0.2) between the proposed system response and actual ICP values.

### Regression Analysis from ML Models

This section presents the results of ML-powered regression analysis performed on prime features shortlisted by the proposed OSS for testing data. A comparison of different ML algorithms described in Table 3 for ICP monitoring using **Sensor A** is shown in Fig. 4. It can be visualized from Fig. 4a that the MAE and RMSE values for Gaussian Process Regression 2 with preset Matern 5/2 GPR have the best performance in this case. The prediction speed (obs/sec) v/s training time (sec) for these ML regression models is shown in Fig. 4b, whereas the coefficient of determination ($R^2$) values are presented in Fig. 4c.

After carefully evaluating all regression models described in Table 3 for the proposed microwave sensors, the following observations were noted:

- For Sensor A, regression model 17: Gaussian Process Regression 2 with Matern 5/2 GPR, performs optimally (MAE: 0.3305, RMSE: 0.4536, $R^2$= 0.99911).
- For Sensor B, regression model 19: Gaussian Process Regression 4 with rational quadratic GPR has the best performance (MAE: 0.1656, RMSE: 0.2209, $R^2$= 0.99984).
- Regression Model 24: Neural Network 5, i.e., trilayered neural network with MAE: 0.2336, RMSE: 0.3314, $R^2$= 0.99965, performs best for Sensor C.
- Similarly, for Sensor D, regression Model 24: Neural Network 5, i.e., trilayered neural network with MAE: 0.1637, RMSE: 0.2841, $R^2$= 0.99958 is optimal.
- Model 5: Tree 1 (fine tree) with MAE: 0.3220, RMSE: 0.4307, $R^2$= 0.99798 has best performance for Sensor E.
- In the case of Sensor F, regression Model 24: Neural Network 5, i.e., trilayered neural network with MAE: 0.1030, RMSE: 0.1613, $R^2$= 0.99986 is the best-performing model.

The average prediction speed of these optimized models is found to be 9899.7952 observations/second, with an average training time of 2.498 s. The plots of predicted ICP v/s true ICP for best performing regression models for each proposed microwave sensor are shown in Fig. 5. These results are plotted using testing data as described in Sect. . It can be observed from Fig. 5 that the predicted ICP from the proposed system closely follows the actual ICP. The distance between antennas ($d_A$) is fixed to 2 cm in this case, but the results are also evaluated for other cases of $d_A$ and are found to be consistent with the presented results. Figure 6 shows the performance comparison of different microwave sensors for ICP monitoring using optimized ML algorithms. It can be visualized from Fig. 6a that the MAE for all the proposed microwave sensors is below 0.33, which showcases the utility of the proposed ML-driven microwave system for non-invasive ICP monitoring. On the other hand, the RMSE is also close to MAE with a maximum value of 0.4536, which suggests that the errors in ICP monitoring are relatively uniform and the impact of outliers on the system’s performance is negligible. Therefore, it can be concluded that the proposed system is likely to make consistent predictions without large deviations with all the studied antenna sensors.

The prediction speed and training time for different microwave sensors are presented in Fig. 6b. It can be visualized from Fig. 6b that the prediction speed of **Sensor A-F** are 7609.95 $\times 10^4$, 11247.6 $\times 10^4$, 5331.77 $\times 10^4$, 8143.88 $\times 10^4$, 15965.5 $\times 10^4$, 11200.1 $\times 10^4$ observations/sec. The coefficient of determination ($R^2$) plot in Fig. 6c showcases the excellent behavior of ML models in explaining the variability in the dependent variable. A high value ($R^2 \approx$0.99) suggests goodness of fit for the ML models. The box plot showing residuals v/s proposed microwave sensors is shown in Fig. 6d, which supports the current discussion. Although the phantom model proposed in this work is meticulously designed to emulate the dielectric properties of a human head, the true dynamics inside the brain are more intricate, owing to physiological compensatory mechanisms, cerebral blood flow modulation, and cerebrospinal fluid circulation. To overcome this limitation, future studies will undertake a comprehensive characterization to study the dynamics of the brain and ICP.

## Conclusions

This study demonstrates the feasibility of an ML–driven microwave system for qualitative and non-invasive monitoring of ICP using realistic head phantom experiments. The proposed system is free from complications related to invasive and implantable devices and thus does not limit its usage to only clinical conditions, making it suitable for wearable applications and prolonged measurements. Numerous simulation and measurement trials on realistic head phantom models showcase a strong analogy between the proposed sensor response and actual pressure measured by invasive sensors. Carefully selected key statistical parameters extracted from microwave sensor response are interfused for ICP monitoring, which showcase a high correlation $\approx 0.9$ with invasive ICP. In only 1.23% of the total test cases, no correlation is observed between microwave sensor response and invasive ICP. A maximum MAE and RMSE of 0.33 and 0.4536 for all microwave sensors highlights the efficiency of the proposed system for ICP monitoring with negligible outliers. These findings represent a proof-of-concept with substantial potential for practical healthcare applications after thorough assessment in animal studies and clinical trials. Future investigations will provide a comprehensive evaluation of ICP waveform morphology and its correlation with the cardiac cycle using deep learning techniques. The shielding effect of the skull, the effect of hair, inter-subject variability, motion artifacts, cable strain, and environmental factors, including sweat & temperature, and interference in complex clinical environments are the main challenges that will be tackled in future studies.

## Materials and methods

**Fig. 7:**
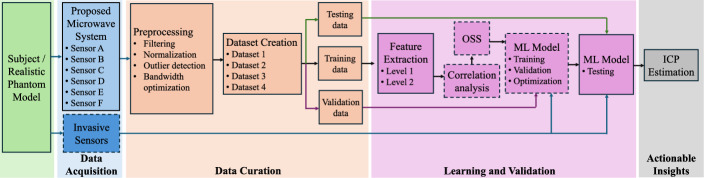
Block diagram depicting data flow in the proposed ML-driven microwave system for noninvasive monitoring of ICP

The proposed system for ICP monitoring consists of small, planar sensors placed on the scalp of the subject or phantom under observation. The proposed system is tested for its robustness by further placing the sensors 1–2 mm away from the scalp. This ensures that marginal errors due to the improper placement of sensors have a negligible effect on the estimated ICP values. The first step is data acquisition, wherein the input reflection coefficients ($S_{XX}$) and reverse transmission coefficient ($S_{XY}$) from different microwave sensors are collected and stored. In addition, invasive sensors are placed inside the skull phantom to measure absolute values for intracranial pressure and temperature. Thereafter, data curation is performed, followed by learning and validation of the proposed ML algorithm. Finally, ICP values are estimated as an outcome of this proposed system as actionable insights. It is important to note that the data from sensors placed inside the skull phantom is used only for the verification of results from the proposed ML-powered microwave system. The block diagram depicting data flow in the proposed system for noninvasive monitoring of ICP is shown in Fig. 7. A detailed description of each sub-block of the proposed system is given in the following subsections.

**Fig. 8:**
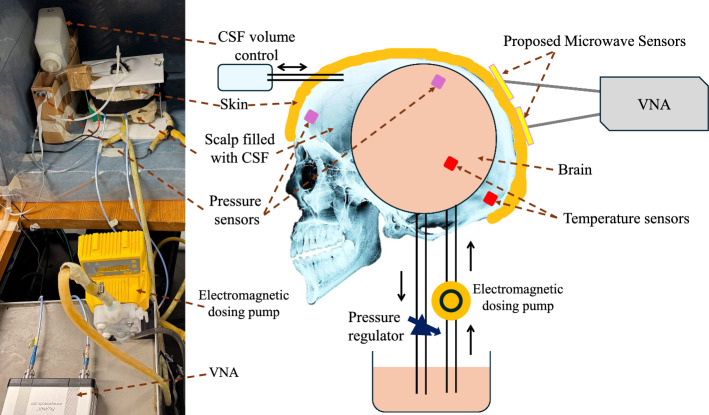
Schematic illustration of developed realistic head phantom with skin, skull, CSF, and brain phantoms (right) and optical photograph of the actual head phantom (left)

### Experimental design

The experimental design for this work is composed of the realistic human head phantom model and measurement setup, including a vector network analyzer (VNA), pumping module, and invasive temperature & pressure sensors for cross-verification of results. The schematic diagram illustrating the developed phantom model and its optical photograph is shown in Fig. 8. The phantom model comprises multiple components, including CSF and brain phantoms encased within an epoxy-based skull phantom, which is further enveloped by a skin phantom. The geometry of epoxy-based skull phantom is derived from CT scans of the average human male head [23]. The skull phantom is fixed in an upright position using a stand. The skull phantom is covered by a skin phantom developed from distilled water, gelatin, sunflower oil, and dishwashing liquid as described in [24]. The skull is filled partially with CSF-mimicking liquid phantom [25]. The CSF volume in the layer between the skull and brain is implemented in this phantom using a flow control mechanism that allows the CSF to flow in and out of the skull phantom in relation to brain phantom pulsations.

A nonporous and flexible pouch positioned within the skull phantom and connected to a programmable electromagnetic dosing pump via a two-pronged hose is used as a storage tank for the brain phantom. When inflated, this pouch replicates the dimensions of an average human brain. A liquid brain phantom prepared by mixing propylene glycol and distilled water in a 1:10 ratio is pumped into the flexible pouch using the programmable electromagnetic dosing pump [26]. The pressure inside the brain phantom is regulated using a pressure regulator connected to the other end of a two-pronged hose. The phantom materials used in this study are selected to correspond with the dielectric properties of human head tissues. The dielectric properties of these phantoms, including permittivity ($\epsilon _r$) and conductivity ($\sigma$), are measured using an open-ended coaxial dielectric probe DAK 3.5 (SPEAG) and validated with biological tissues. The measured results for different phantoms, including skin, skull, CSF, and brain, at 4 GHz are presented in Table 1. It can be observed from Table 1 that the values of ($\epsilon _r$) and ($\sigma$) for the brain and skin phantom are matching with their biological counterparts. The use of epoxy for the skull phantom results in a difference between the skull phantom and the actual skull, which shows a lower $\epsilon _r$ and higher $\sigma$ values. This difference is compensated to some extent by carefully selecting a CSF phantom with higher $\epsilon _r$ and lower $\sigma$ values than biological CSF.

**Table 1:** Dielectric properties ($\epsilon _r$ and $\sigma$) of phantoms and biological tissue at 4 GHz [27, 28]

Tissue	Phantom		Biological tissue	
	$\epsilon _r$	$\sigma$ (S/m)	$\epsilon _r$	$\sigma$ (S/m)
Skin	35.4	2.61	36.6	2.34
Skull	5.3	2.57	13.7	1.06
CSF	81.3	3.52	63.7	5.2
Brain	40.4	2.73	40.5	2.61

The microwave-based ICP sensors (presented in Sect. ) are deployed on the skin phantom and connected to a VNA (dynamic range=124dB, RMS trace noise=$5\times 10^{-3}$dB) using coaxial cables. The VNA is calibrated from 2 to 6 GHz (2001 points) using a standard calibration kit. The VNA is programmed to operate in autonomous mode synchronized with the beats of an electromagnetic dosing pump. Two board-mounted pressure sensors: SSCDRRN005PDAA5 from Honeywell Sensing and Productivity Solutions, and two negative temperature coefficient (NTC) thermistors are used as invasive sensors to capture real-time pressure and temperature values, respectively, inside the brain phantom and between the brain and skull phantom as shown in Fig. 8. The pump, VNA, and invasive sensors are operated using a LabVIEW program, and the timing details of each measurement are recorded for synchronization purposes.

The microwave antennas proposed for ICP monitoring in this study are presented in the following subsection. Extensive measurements are performed on the developed phantom model to establish an analytical framework for ICP monitoring. Every trial started with pumping brain phantom liquid gradually into the brain phantom pouch. The dosing pump is operated at 60 beats per minute until the pressure inside the brain phantom reaches 60 mmHg. The reference data are recorded using 5 KHz sampling rate by commercial pressure sensors placed inside the skull. The VNA operates at 3 Hz in synchronization with pump beats. The room temperature of experiential design is maintained at 23$^0C$.

**Fig. 9:**
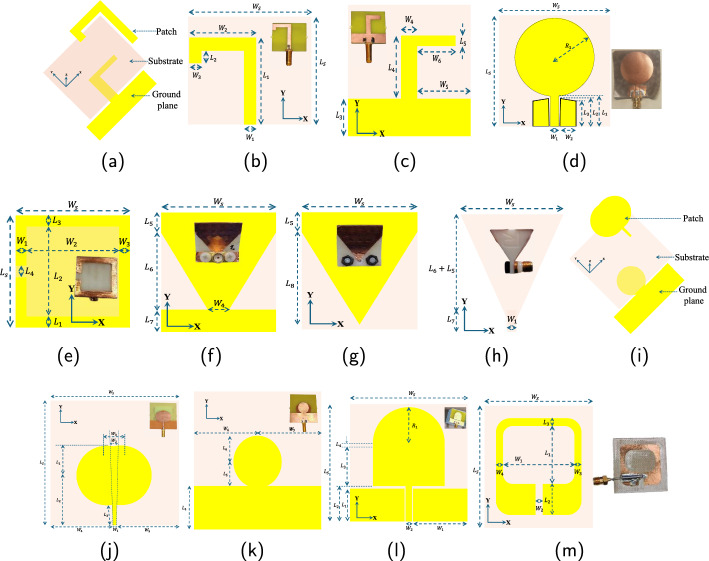
Proposed microwave sensors for ICP monitoring, (a),(b),(c): 3D schematic illustration of patch, substrate, and ground plane; detailed front and back view of sensor ${\textbf {A}}$, respectively; (d): schematic illustration of Sensor ${\textbf {B}}$; (e), (f),(g), (h): top, left, right, and side views of proposed sensor ${\textbf {C}}$, (i),(j),(k): 3D schematic illustration; detailed front and back view of sensor ${\textbf {D}}$, respectively, (l),(m): schematic illustration of Sensor ${\textbf {E}}$ and ${\textbf {F}}$, respectively. The optical photographs of the fabricated sensors are given along with the schematics

### Proposed microwave system

This study proposes and tests six different microwave sensors in two configurations. These sensors are optimized in terms of size, operating frequency, and gain to make them suitable for biomedical sensing applications. The first configuration includes placing antennas on the same side of the skull with horizontal separation $d_A$ = 5, 10, and 20 mm. The results for $d_A>$ 20 mm are also promising, but abandoned for system size optimization and practical applications of the proposed sensor for wearable use. The two antennas are placed diagonally on opposite sides of the skull in the second configuration. It is to be noted that the measurements and simulations were also conducted for other cases, i.e., intermediate antenna distances ($d_A$) between 5–10 mm and 10–20 mm, and different antenna locations on the skull phantom; these results are not plotted because of their visual redundancy. The magnitude and phase of input reflection coefficients ($S_{XX}$) and reverse transmission coefficient ($S_{XY}$) are recorded during each measurement. The detailed dimensions of the sensor design are given in Table 2. The subsequent subsections present the details of different sensor designs undertaken in the study.

#### Sensor ${\textbf {A}}$

The first antenna, Sensor ${\textbf {A}}$, is printed on a low-cost FR4 substrate and has small dimensions of 25$\times$30.3$\times$1.6 mm. The design was originally presented in [29] and further developed using a cavity-backed approach for in-body communication. The 3D schematic illustration of the proposed sensor for ICP monitoring is shown in Fig. 9a with a detailed front and back view in Fig. 9b, c, respectively. The optical photographs of the fabricated sensors are given along with the schematics. The radiating element on one side of the substrate constitutes a close inverted L shape of width 3 mm. The ground plane on the other side consists of a rectangular element of size 7.14$\times$30.3 mm and an inverted L shape. The sensor is fed using a microwave feed line of width 3 mm, which is connected to the coaxial cable using a standard SMA connector and has a free space measured impedance bandwidth of 3.75$-$4.50 GHz.

#### Sensor ${\textbf {B}}$

To further test the consistency of the devised ICP monitoring method, a thin, flexible monopole antenna is proposed. The antenna is a modification of the flexible antenna designed in [30]. The wideband antenna is fabricated on a 40 $\times$40 mm Rogers5880 substrate of thickness 0.125 mm and operates at 2–10 GHz. Figure 9d shows the schematic of sensor ${\textbf {B}}$ along with the optical photograph of the fabricated sensor.

#### Sensor ${\textbf {C}}$

Sensor ${\textbf {C}}$ is a rectangular horn antenna with small dimensions of 20$\times$21 mm. The design of the proposed sensor ${\textbf {C}}$ for ICP monitoring is shown in 9e, top view, f, g detailed left and right view, and h side view, along with an optical photograph of the fabricated sensor. The design of the antenna is based on a mini-horn antenna originally presented in [31] made by 3D-printed polylactic acid. For improving the bio-matching, 15 cylindrical holes of diameter 2 mm are drilled into the antenna and filled with distilled water.

#### Sensor ${\textbf {D}}$

Antenna ${\textbf {D}}$ has a rectangular-shaped structure printed on FR4 substrate of thickness 0.8 mm with size 47.5$\times$47.5 mm [32]. The radiating patch consists of two concatenated circles fed by a microstrip line of width 1.5 mm. An overlapping circle and rectangular shape create the ground plane. The 3D schematic illustration of the proposed sensor ${\textbf {D}}$ is shown in Fig. 9i. The front and back views of sensor ${\textbf {D}}$ are shown in Fig. 9j, k, respectively, along with optical photographs of fabricated sensors. This sensor has a resonant frequency of 3.88 GHz, with a bandwidth of 3.47$-$4.27 GHz and a return loss of $-$32.4 dB.

#### Sensor ${\textbf {E}}$

Sensor ${\textbf {E}}$ is a low UWB antenna with an operating bandwidth of 3.62$-$4.73 GHz and a gain of 3.25 dBi. The antenna is printed on a low-cost FR4 substrate of thickness 1.6 mm with dimensions 36$\times$36 mm$^2$ [33], as shown in Fig. 9l, along with the optical photograph of the fabricated sensor.

#### Sensor ${\textbf {F}}$

Sensor *F* has dimensions of $38\times 39\times 1.6 mm^3$ with a modified disk structure as a radiating patch. The antenna is fabricated on a low-cost FR4 substrate. The detailed dimensions of Sensor *F* are given in Table 2, and the schematic of the design is shown in Fig. 9m.

**Table 2:** Dimensions, substrate material, and bandwidth (BW) of different microwave sensors utilized in this study for ICP monitoring

Sensor A (Substrate: FR4, BW: 3.75–4.5 GHz)							
Parameter	$L_S$	$H_S$	$L_1$	$L_2$	$L_3$	$L_4$	$L_5$
Value	25	1.06	20.14	2.09	7.14	12	2
Parameter	$W_S$	$W_1$	$W_2$	$W_3$	$W_4$	$W_5$	$W_6$
Value	30.3	3	16.04	3	4	13.15	9.05
Sensor B (Substrate: Rogers5880, BW: 2–10 GHz)							
Parameter	$L_S$	$H_S$	$L_1$	$L_2$	$L_3$		
Value	40	0.125	10.88	10	9.1		
Parameter	$W_S$	$W_1$	$W_2$	$R_1$			
Value	40	3	5.7	14			
Sensor **C** (Substrate: 3D-printed polylactic acid, BW: 1.4–8.5 GHz)							
Parameter	$L_S$	$L_1$	$L_2$	$L_3$	$L_4$	$L_5$	$L_6$
Value	22	2	18	2	2	4	16
Parameter	$W_S$	$W_1$	$W_2$	$W_3$	$W_4$	$L_7$	$L_8$
Value	22	2	18	2	4	4.5	19
Sensor **D** (Substrate: FR4, BW: 2.5–4.5 GHz)							
Parameter	$L_S$	$H_S$	$L_1$	$L_2$	$L_3$	$L_4$	
Value	47.5	0.8	9	21.5	14.02	14.9	
Parameter	$W_S$	$W_1$	$W_2$	$W_3$	$W_4$	$W_5$	
Value	47.5	1.5	3	8	22.75	23.75	
Parameter	$L_5$	$L_6$	$W_6$	$W_7$			
Value	8.4	9	22.75	23.75			
	Sensor **E** (Substrate: FR4, BW: 3.62–4.73 GHz)						
Parameter	$L_S$	$H_S$	$L_1$	$L_2$	$L_3$	$L_4$	$R_1$
Value	36	1.6	10	10.8	12	1.3	11
Parameter	$W_S$	$W_1$	$W_2$				
Value	36	17.5	2				
	Sensor **F** (Substrate: FR4, BW: 3.47–4.27 GHz)						
Parameter	$L_S$	$H_S$	$L_1$	$L_2$	$L_3$		
Value	38	1.6	15	8	2		
Parameter	$W_S$	$W_1$	$W_2$	$W_3$	$W_4$		
Value	39	20	2	2	2		

### Signal acquisition and curation

The procedure for data collection, preprocessing, and data set creation is as follows.

#### Data collection

In the first step, microwave sensors are applied to the phantom setup as described in Sect. . The magnitude and phase of input reflection coefficients (${\varvec{{{S}}}}^{i,j,f}_{XX}$) and reverse transmission coefficient (${\varvec{{{S}}}}^{i,j,f}_{XY}$) of $i^{th}$ sensor ($i \in \{A, B, C, D, E, F\}$) from $j^{th}$ measurement trial ($j \in \{1, 2, \cdots Tr\}$) are recorded. Here, *Tr* is the total number of trials conducted for a particular sensor, $f \in \mathbb {B}$ corresponds to instantaneous frequency, and $\mathbb {B}$ is the bandwidth of the microwave system. Apart from this, actual pressure (${\varvec{{{p}}}}_T$) and temperature (${\varvec{{{t}}}}_T$) data from invasive sensors is also recorded. The triggering signals utilized to excite the pump and VNA are recorded for synchronization purposes.

There are three main challenges identified that may affect the quantitative assessment of ICP based on the measured microwave signals. These issues include variations in head shape, differences in skull geometry & tissue thickness, and sensor measurement positions. To address the effect of antenna separation, all six sensors are tested with different values of inter-antenna distance, i.e., $d_A$ = 5, 10, 20, 30 mm, and antennas on opposite sides of the head phantom. The horizontal distance between the sensors is varied after each measurement to study the sensing characteristics. Furthermore, the issue of skull geometry and variation in head shape is partially resolved by placing the antennas at different locations around the head model and repeating the measurements five times for each antenna configuration. The results obtained from these trials were averaged to remove randomness in the readings and ensure repeatability. The standard error of the mean (SEM) is calculated between five trials for each antenna configuration and was found to be < 1 mmHg for all cases. The proposed system is also tested for its robustness by further placing the sensors 1–2 mm away from the scalp. This ensures that marginal errors due to the improper placement of sensors have a negligible effect on the estimated ICP values.

#### Pre-processing

In the next step, the data collected from measurements is pre-processed. This includes removing initial samples, wherein no change is observed in the pressure values. Furthermore, normalization and outlier detection are applied to each data string to ensure consistent transformation of all data strings. The process of filtering, normalization, and outlier detection is conducted using the function $\zeta _1$ represented as

1 $$\begin{aligned} \mathbb {S}_1^{i,j,f} = \zeta _1[\mathbb {S}^{i,j,f}], \end{aligned}$$

where $\mathbb {S}^{i,j,f} \in \{|{\varvec{{{S}}}}^{i,j,f}_{XX}|, |{\varvec{{{S}}}}^{i,j,f}_{XY}|, \angle {\varvec{{{S}}}}^{i,j,f}_{XX}, \angle {\varvec{{{S}}}}^{i,j,f}_{XY}\}$. Next, the signal is band-limited in frequency to decrease computation time and realize near-real-time operation. This band-limiting operation is based on a statistical operation of finding the frequency band showcasing maximum variation in ${\varvec{{{S}}}}^{i,j,f}_{XX}$ and ${\varvec{{{S}}}}^{i,j,f}_{XY}$. This results in bandwidth optimization as the complete operational bandwidth of the microwave system is not required, and only the most useful frequency band of operation is selected. The process starts by first selecting the location ($loc^f_{S_{XX}}$) and values ($val^f_{S_{XX}}$) for minima of ${\varvec{{{S}}}}^{i,j,f}_{XX}$, mathematically represented as:

2 $$\begin{aligned} \left[ loc^f_{S_{XX}},val^f_{S_{XX}}\right] = {\textbf {MIN}}\left[ \left| {\varvec{{{S}}}}^{i,j,f}_{XX}\right| \right] . \end{aligned}$$

Thereafter, the optimal frequency ($f_{S_{XX}}^{opt}$) is selected which represents maximum deviations in $val^f_{S_{XX}}$ as

3 $$\begin{aligned} f_{S_{XX}}^{opt} = {\textbf {MAX}}\left[ {\textbf {MAX}}\left[ val^f_{S_{XX}}\right] - {\textbf {MIN}}\left[ val^f_{S_{XX}}\right] \right] . \end{aligned}$$

Finally, the optimal bandwidth ($\mathbb {B}_{S_{XX}}^{opt} \subset \mathbb {B}$) is calculated around $f_{S_{XX}}^{opt}$ as

4 $$\begin{aligned} \mathbb {B}_{S_{XX}}^{opt} = \left[ f_{S_{XX}}^{opt}-f_{th} : f_{S_{XX}}^{opt}+f_{th}\right] , \end{aligned}$$

where $f_{th}$ is the threshold frequency governing the operational bandwidth of the system and is selected as 50 MHz to capture all significant features and ensure a lightweight algorithm. In addition, the signal is also time-limited to the useful range of values, including readings, where pressure changes gradually from 0 mmHg to 60 mmHg. The process is mathematically represented as

5 $$\begin{aligned} \mathbb {S}_2^{i,j,f} = \zeta _2\bigg [\mathbb {S}_1^{i,j,f}\bigg ]_{f_{S_{XX}}^{opt}-f_{th}}^{f_{S_{XX}}^{opt}+f_{th}}, \end{aligned}$$

where $\zeta _2$ represents the band-limiting operation. To maintain uniformity, the number of pump pulses was also recorded and compared with observed pressure to always maintain the same volume of brain and CSF phantoms inside the skull phantom at the end of each trial. Finally, the antenna responses are co-aligned and synchronized with actual pressure values in time and frequency domains using reference data of VNA and pump triggering.

#### Data set creation

Another distinctive attribute of the proposed algorithm is the development of four different data sets from the measured data. In the second step, the data collected during each trial is divided into four data sets. The description for these data sets is as follows:

- The first data set, denoted by ${\textbf {Ds}}_1$, contains the complete data from a sensor measured during an individual trial.
- The first 80% data samples are taken in second data set denoted by ${\textbf {Ds}}_2$.
- In third data set ${\textbf {Ds}}_3$, the last 80% data samples are taken.
- Finally, fourth data set (${\textbf {Ds}}_4$) is generated by taking middle 80% data samples from 10% to 90% of the data of a trial.

The overall data set created by this process is denoted by $\mathbb {D}_k \in \{{\textbf {Ds}}_1, {\textbf {Ds}}_2, {\textbf {Ds}}_3, {\textbf {Ds}}_4 \}$. The inclusion of this step in the proposed algorithm grants additional stability and robustness to the ICP monitoring process. Thereafter, in the next step, the measurement data are divided randomly into training (80%), testing (10%), and validation data sets (10%), using function $\zeta _3$ denoted by $\mathbb {T}_l$. This data splitting is performed carefully to ensure that the data samples among the training and test data sets are uncorrelated. For this, samples from one trial are used either as training or testing data sets, whereas an independent trial is performed for the creation of its counterpart. This is made possible as measurements for each antenna configuration for all six microwave sensors were repeated 5 times to generate independent samples. This ensures that the system is always tested on unseen data from an independent trial.

### Learning and validation

The next stage is the proposed ML-powered quantitative method for ICP monitoring. This includes a unique two-level feature extraction module followed by training, validation, and optimization of the ML model.

#### Feature extraction

Following data set creation and dividing the total data randomly into testing, training, and validation data sets, feature extraction is performed. The features extracted in this study are divided into two levels. Level 1 features include the value and location of the maxima and minima of magnitude and phase of antenna reflection coefficients in the frequency domain. Apart from this, the Area Under the Curve (AUC) is also calculated for all these parameters. It is observed that AUC provides a unique single-digit characteristic of signal shape, which is easy to analyze and process. Level 2 features include calculating Level 1 features from higher level parameters, such as differential multi-static-data matrix (DMDM), root mean square (RMS)-based MDM (RMS–MDM) matrix, group delay distortion (GDD), and percent amplitude of fluctuation (PerAF). A detailed explanation of these higher level parameters is given in Appendix A. The process of feature extraction is mathematically represented as

6 $$\begin{aligned} {[}\psi _1,\omega _1] =\zeta _4[\mathbb {T}_{l}], \end{aligned}$$

where $\psi _1$, $\omega _1$ are **Level 1** features representing the value and location, respectively, calculated by applying function $\zeta _4$ on testing, training, and validation data sets. The function $\zeta _4$ is defined as $\zeta _4 \in \left[ {\textbf {MAX}}[\cdot ], {\textbf {MIN}}[\cdot ]\right]$. The features $\psi _1, \omega _1$ are calculated for both magnitude and phase values. In addition, another **Level 1** feature $\psi _2$ is calculated as

7 $$\begin{aligned} \psi _2 = AUC[\mathbb {T}_{l}], \end{aligned}$$

where *AUC* represents the function calculating the area under the curve. Next, the **Level 2** features for $MDM_D$, $MDM_{RMS},$, *PerAF* and *GDD* are calculated as

8 $$\begin{aligned} {[}\psi _3,\omega _3] = \zeta _4[\zeta _5[\mathbb {T}_{l}]], \end{aligned}$$

where $\psi _3$ and $\omega _3$ represent the value and location-based features obtained by applying function $\zeta _4$ on the output of function $\zeta _5$, which is defined as: $\zeta _5 \in [MDM_D, MDM_{RMS}, PerAF, GDD]$. Similar to (7), the AUC parameters are calculated for this case as

9 $$\begin{aligned} {[}\psi _4] = AUC[\zeta _5 [\mathbb {T}_{l}]], \end{aligned}$$

An important point to note is that both the phase and magnitude of $S_{XX}$ and $S_{XY}$ are utilized in this study for analysis, presenting a strong framework for ICP monitoring.

**Table 3:** Details of ML models used for ICP monitoring

No	Model	Preset
1	LR 1	Linear
2	LR 2	Interactions linear
3	LR 3	Robust linear
4	Tree 1	Fine tree (leaf size=4)
5	Tree 2	Medium tree (leaf size=12)
6	Tree 3	Coarse tree (leaf size=36)
7	SVM 1	Linear SVM
8	SVM 2	Quadtratic SVM
9	SVM 3	Cubic SVM
10	SVM 4	Fine gaussian SVM
11	SVM 5	Medium gaussian SVM
12	SVM 6	Coarse gaussian SVM
13	Ensemble 1	Boosted trees
14	Ensemble 2	Bagged trees
15	GPR 1	Squared exponential
16	GPR 2	Matern 5/2
17	GPR 3	Exponential
18	GPR 4	Rational quadratic
19	NN 1	Narrow NN (size=10)
20	NN 2	Medium NN (size=25)
21	NN 3	Wide NN (size=100)
22	NN 4	Bilayered NN (size=10)
23	NN 5	Trilayered NN (size=10)
24	Kernel 1	SVM kernel
25	Kernel 2	LSR kernel

**Algorithm 1:**
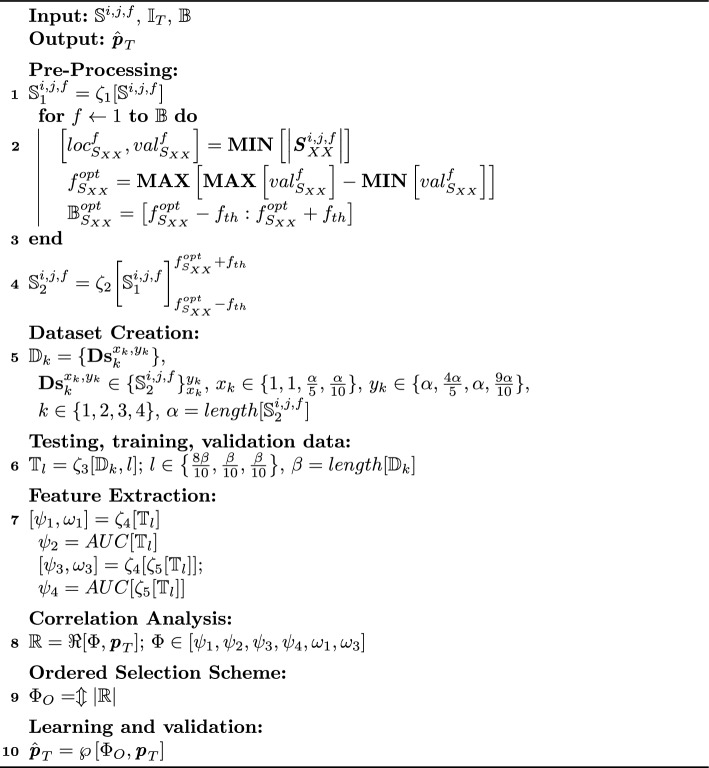
Proposed algorithm for noninvasive ICP monitoring

#### Correlation analysis

Before supplying the data to ML models, correlation analysis is conducted on the features extracted from the measured data to find an association between sensor response and actual pressure values (${\varvec{{{p}}}}_T$). Pearson correlation coefficients are calculated with $p<$ 0.001 as

10 $$\begin{aligned} \mathbb {R} = \Re [\Phi ,{\varvec{{{p}}}}_T], \end{aligned}$$

where $\mathbb {R}$ is the Pearson correlation coefficients and $\Phi \in [\psi _1, \psi _2, \psi _3, \psi _4, \omega _1, \omega _3]$ represents set of extracted features. The results of the correlation analysis are presented using a Sankey diagram. Following extensive correlation computations on measured data from all six sensors in numerous trials, it is observed that using a very large number of statistical features increases the computational complexity of the ML models for ICP monitoring. The following subsection presents a resolution to this problem.

#### Ordered selection scheme (OSS)

To support real-time operation and a lightweight algorithm, we propose a unique Ordered Selection Scheme (OSS) to restrict the number of features without affecting the performance of ICP monitoring. The proposed OSS scheme is inspired by information theory, wherein only the strongest attributes are selected to estimate a given problem [34]. These attributes are selected by ordering all the attributes in decreasing order of strength. The correlation between true pressure values (${\varvec{{{p}}}}_T$) from invasive sensors and feature set ($\Phi$) developed using Level 1 and Level 2 features is taken as the measure of strength for OSS scheme as

11 $$\begin{aligned} \Phi _O = \Updownarrow \left| \mathbb {R} \right| , \end{aligned}$$

where $\Updownarrow$ and $|\cdot |$ represents sorting from largest to smallest and absolute operations.

After sorting the features, only the strongest features are selected for further analysis. This selection process is governed by the correlation ($\mathbb {R}$) with a threshold set to $\mathbb {R}>0.8$. Pearson’s coefficient of correlation ($\mathbb {R}$) is calculated for this analysis, with statistical significance of correlation values (*p* values) defined as $p < 0.05$. All statistical computations were performed in MATLAB R2024a using the **corr** function. OSS ensures that the selected features are sufficient for accurate monitoring of ICP using the proposed setup with every antenna in all placement strategies ($d_A$). It is found that only twelve features are sufficient for accurate monitoring of ICP using the proposed setup. These features are: *a*:$min[|{\varvec{{{S}}}}^{i,j,f}_{XX}|]$, *b*:$min[|{\varvec{{{S}}}}^{i,j,f}_{XY}|]$, *c*:$AUC[|{\varvec{{{S}}}}^{i,j,f}_{XX}|]$, *d*:$AUC[|{\varvec{{{S}}}}^{i,j,f}_{XY}|]$, *e*:$AUC[GDD_{\angle {\varvec{{{S}}}}^{i,j,f}_{XX}}]$, *f*:$AUC[GDD_{\angle {\varvec{{{S}}}}^{i,j,f}_{XY}}]$, *g*:$AUC[MDM_D]$, *h*:$AUC[MDM_{RMS}]$, *i*:$PerAF_{|S_{XX}|}$, *j*:$PerAF_{|S_{XY}|}$; *k*:$PerAF_{\angle {\varvec{{{S}}}}^{i,j,f}_{XX}}$, *l*:$PerAF_{\angle {\varvec{{{S}}}}^{i,j,f}_{XY}}$. If the number of features is further increased, no significant improvement in system performance is observed. It is important to note that the proposed OSS scheme and correlation analysis are only required once for selecting the optimal features.

#### Proposed ML model

After correlation analysis and OSS, the extracted features shortlisted by OSS ($\Phi _O$) are fed to regression-based ML models. This ensures autonomic and quantitative monitoring of ICP $\hat{{\varvec{{{p}}}}}_T$ from features $\Phi _O$. The process can be mathematically represented as:

12 $$\begin{aligned} \hat{{\varvec{{{p}}}}}_T = \wp [\Phi _O,{\varvec{{{p}}}}_T], \end{aligned}$$

where $\wp$ represents the ML regression operator. To test the performance of various standard regression operators proposed in the literature, a total of 25 models are tested. These models belong to different families, including linear regression, tree models, Support Vector Machine (SVM), Ensemble-based regression, Gaussian Process regression, Neural Networks (NN), and kernel-based regression. The details of ML models used for ICP monitoring are given in Table 3.

These models are trained using training data (80% of total samples) and then validated using a validation data set (10% of total samples). After numerous trials on all six proposed microwave sensors and antenna placement strategies, the best-performing ML model is shortlisted and optimized. This selection is based on standard performance parameters of ML models: Mean Absolute Error (MAE), Root Mean Square Error (RMSE), Prediction Speed (observation/sec), Training Time (sec), and coefficient of determination ($R^2$). The system does not involve hyperparameter tuning, and all model evaluations are performed in the outer folds. To remove the problem of overfitting, fivefold cross-validation is utilized in the system. The final step of proposed ML-powered ICP monitoring system includes testing the shortlisted ML model on testing data (10% of total samples), which provides actionable insights of the system in the form of ICP values. The overall process and data flow in the proposed ML-driven microwave system for noninvasive monitoring of ICP is presented as a block diagram in Fig. 7. The proposed algorithm for noninvasive ICP monitoring is presented in Algorithm 1.

## Data Availability

No data sets were generated or analysed during the current study.

## Multi-static-Data Matrix (MDM)

A differential Multi-static-Data Matrix (DMDM) approach is employed to extract higher level features and eliminate the effect of mutual coupling. The MDM matrix $MDM^{i_1,i_2}$ is composed of antenna response between $i_1^{th}$ transmit and $i_2^{th}$ receive antenna calculated as [35]

A1 $$\begin{aligned} M^{i_1,i_2} =\begin{bmatrix} |{\varvec{{{S}}}}_{i_1i_1}| & |{\varvec{{{S}}}}_{i_1i_2}| \\ |{\varvec{{{S}}}}_{i_2i_1}|& |{\varvec{{{S}}}}_{i_2i_2}| \end{bmatrix} \end{aligned}$$

where $|{\varvec{{{S}}}}_{XX}|$ and $|{\varvec{{{S}}}}_{XY}|$ represent the input reflection coefficients and reverse transmission coefficient of the antenna, respectively. The DMDM is calculated as [36]

A2 $$\begin{aligned} MDM_{d} = MDM_t - MDM_c, \end{aligned}$$

where $MDM_t$ and $MDM_c$ are the MDM matrix with target and clutter (no target), respectively. The singular value decomposition (SVD) of $MDM_d$ results in

A3 $$\begin{aligned} MDM_{d} = \kappa \vartheta \varkappa ^*, \end{aligned}$$

where $\kappa$ and $\varkappa$ are the singular vectors and the diagonal elements of $\vartheta$ represent singular values of $MDM_d$. The dominant singular value $\vartheta _d = max[\vartheta ]$ is further utilized to represent the overall sensor response in a particular scenario.

## Root mean square (RMS)-based MDM (RMSMDM)

Another method based on the differential matrix of Root Mean Square (RMS)-based MDM is utilized to get more insights into the effect of antenna configuration and location. The RMS–MDM matrix is calculated as

B4 $$\begin{aligned} MDM_{RMS}= \sqrt{MDM_t^2 - MDM_c^2}, \end{aligned}$$

The dominant singular value of $MDM_{RMS}$, i.e., $\vartheta _{RMS}$, is also used as a feature for further investigations.

## Group delay distortion (GDD)

GDD is calculated from the phase response of the proposed microwave sensor. GDD is a function of reflection group delay $\left( \frac{\partial \angle {\varvec{{{S}}}}}{\partial f}\right)$ which is calculated by differentiating the phase response of the sensor with frequency [15]. We define GDD as the difference between reflection group delay with and without target and is mathematically calculated as [15]

C5 $$\begin{aligned} GDD = \frac{\partial \angle {\varvec{{{S}}}}_t}{\partial f} - \frac{\partial \angle {\varvec{{{S}}}}_c}{\partial f}, \end{aligned}$$

where $\angle {\varvec{{{S}}}}_t$ and $\angle {\varvec{{{S}}}}_c$ are the sensor responses with target and clutter (no target), respectively.

## Percent amplitude of fluctuation (PerAF)

The PerAF is utilized to measure the percentage of fluctuations in the antenna response relative to the mean antenna response for each instance in time and averaged across the whole time series [37]. The scale-independent PerAF is calculated as

D6 $$\begin{aligned} PerAF = \frac{1}{N} \sum _{n=1}^N \left| \frac{\mathbb {S}_n - \mu }{\mu }\right| \times 100, \end{aligned}$$

where $\mathbb {S}_n$ is the sensor response at time instance *n*, $\mu$ is the mean of $\mathbb {S}_n$ across all timepoints (*N*).
